# Identification of a Novel Small Cysteine-Rich Protein in the Fraction from the Biocontrol *Fusarium oxysporum* Strain CS-20 that Mitigates *Fusarium* Wilt Symptoms and Triggers Defense Responses in Tomato

**DOI:** 10.3389/fpls.2015.01207

**Published:** 2016-01-07

**Authors:** Larisa A. Shcherbakova, Tatyana I. Odintsova, Alexander A. Stakheev, Deborah R. Fravel, Sergey K. Zavriev

**Affiliations:** ^1^Laboratory of Physiological Plant Pathology, All-Russian Research Institute of PhytopathologyMoscow, Russia; ^2^Laboratory of Molecular-Genetic Bases of Plant Immunity, Vavilov Institute of General GeneticsMoscow, Russia; ^3^Laboratory of Molecular Diagnostic, M. M. Shemyakin and Yu. A. Ovchinnikov Institute of Bioorganic Chemistry of the Russian Academy of SciencesMoscow, Russia; ^4^Crop Production and Protection, United States Department of Agriculture, Agricultural Research ServiceBeltsville, MD, USA

**Keywords:** *F. oxysporum* strain CS-20, *Fusarium* wilt of tomato, biogenic elicitor, cysteine-rich proteins, induced resistance, biocontrol

## Abstract

The biocontrol effect of the non-pathogenic *Fusarium oxysporum* strain CS-20 against the tomato wilt pathogen *F. oxysporum* f. sp. *lycopersici* (FOL) has been previously reported to be primarily plant-mediated. This study shows that CS-20 produces proteins, which elicit defense responses in tomato plants. Three protein-containing fractions were isolated from CS-20 biomass using size exclusion chromatography. Exposure of seedling roots to one of these fractions prior to inoculation with pathogenic FOL strains significantly reduced wilt severity. This fraction initiated an ion exchange response in cultured tomato cells resulting in a reversible alteration of extracellular pH; increased tomato chitinase activity, and induced systemic resistance by enhancing *PR-1* expression in tomato leaves. Two other protein fractions were inactive in seedling protection. The main polypeptide (designated CS20EP), which was specifically present in the defense-inducing fraction and was not detected in inactive protein fractions, was identified. The nucleotide sequence encoding this protein was determined, and its complete amino acid sequence was deduced from direct Edman degradation (25 N-terminal amino acid residues) and DNA sequencing. The CS20EP was found to be a small basic cysteine-rich protein with a pI of 9.87 and 23.43% of hydrophobic amino acid residues. BLAST search in the NCBI database showed that the protein is new; however, it displays 48% sequence similarity with a hypothetical protein FGSG_10784 from *F. graminearum* strain PH-1. The contribution of CS20EP to elicitation of tomato defense responses resulting in wilt mitigating is discussed.

## Introduction

The vascular wilt pathogen *Fusarium oxysporum* f. sp. *lycopersici* (FOL) is one of the most destructive pathogens of greenhouse and field grown tomatoes (Benhamou et al., 1989; Fravel et al., 2003b; Anitha and Rabeeth, 2009; Kaur et al., 2010). Integrated protection against *Fusarium* wilt includes biological control as an important alternative or component of disease management. This pathogen can be controlled by various microorganisms including nonpathogenic strains of *F. oxysporum*, which are used individually or together with soil bacteria (Fravel et al., 2003b; Silva and Bettiol, 2005; Alabouvette et al., 2009). As with other microbial agents, nonpathogenic *F. oxysporum* strains employ several modes of action contributing to their biocontrol activity and the modes may vary depending on the strain or environment (Fravel et al., 2003b; Alabouvette et al., 2009). For instance, main mode of action of *F. oxysporum* strains C5 and C14 is competition for nutrient sources (Mandeel and Baker, 1991). Well-documented as a protective agent, strain Fo47 inhibits spore germination and germ tube growth of the pathogen (Larkin and Fravel, 1999; Olivain et al., 2006). Strain Fo47 also controls FOL by priming of six genes involving in tomato defense responses (Aime et al., 2013). Another nonpathogenic *F. oxysporum* strain CS-20 does not affect the pathogen directly, but involves plant-mediated mode of action and functions primarily by inducing the disease resistance (Larkin and Fravel, 1999; Panina et al., 2007).

Resistance in plants can be induced by general or/and specific elicitors, which are widely present in both plant pathogenic and beneficial microorganisms. These elicitors can be proteins, peptides, glycoproteins, lipids, oligosaccharides, or polysaccharides. As signaling molecules, elicitor and mediator proteins capable of triggering plant defense responses play often an important role in development of SAR or ISR caused by fungi that control diseases in various plants. For instance, proteins with enzymatic functions, hydrophobines or small avirulence proteins from *Trichoderma* strains elicit an array of plant defense reactions against bacteria and fungi damaging on cucumber, tomato, maize, cotton, and tobacco (Hanson and Howell, 2004; Djonović et al., 2006; Seidl et al., 2006; Hermosa et al., 2012; Freitas et al., 2014; Ruocco et al., 2015). Discovery of proteinaceous elicitors provide new potential tools for crop pathogen control by ecologically sound disease management strategies, including expression of the elicitor genes in transgenic plants (Islam, 2006; Kromina and Dzhavakhiya, 2006).

The strain CS-20 considerably reduces wilt incidence on tomato, as well as on muskmelon and basil. It is effective in sandy, loamy and heavy clay soils, and is able to protect susceptible and resistant tomato cultivars against all three races of the pathogen as well as multiple pathogenic strains of each race (Larkin and Fravel, 1999, 2002; Larkin et al., 1999). These properties make CS-20 very promising as an anti-wilt agent. Knowledge of the modes of action may provide avenues to expand the use of this biocontrol agent. Previous research that was done to clarify mechanisms underlying the plant-mediated biocontrol effect of CS-20 was focused on identification and characterization of genes related to its biocontrol ability to identify differences in gene regulation between CS-20 and pathogenic FOL isolates (Fravel et al., 2003a, 2007, 2008). The work reported here was undertaken to further clarify the plant-mediated mode of action of CS-20 by determining its ability to produce elicitors of defense responses, which are associated with local and/or systemic resistance and result in reduction of wilt severity on tomatoes.

## Materials and Methods

### Production of *F. oxysporum* CS-20 Biomass, Isolation of Fungal Metabolites by Extraction with Buffer Followed by Sephadex Gel Filtration

Strain CS-20 was cultivated in 750 ml Erlenmeyer flasks with 100 ml of liquid Czapek’s medium on an orbital shaker at 220 rpm and 25°C in the darkness for 3 days. Fungal biomass was harvested by vacuum filtration through sterile Miracloth, washed with a threefold volume of sterile distilled water and ground into a fine powder under liquid nitrogen. The powdered biomass was extracted by stirring in 0.05 M sodium phosphate buffer, pH 6.0 with 1 M KCl for 45 min at 4°C (3 ml of the buffer per 1 g air-dried biomass). The homogenate was clarified by centrifugation at 3000 × *g* for 30 min at 4°C. The pellet was discarded, and supernatant was centrifuged at 15000 × *g* for 25 min at 4°C. A double volume of saturated (NH_4_)_2_SO_4,_ was added to the supernatant. After overnight incubation at 4°C, the precipitate was separated by centrifugation at 15000 × *g* for 10 min and dissolved in a minimal volume of double distilled water (ddH_2_O). The dissolved precipitate was either subjected to ultrafiltration (see below) or loaded on a gel filtration column (5 cm × 32 cm) with Sephadex G-50 calibrated against Blue Dextran with molecular weight of 2000 kDa. The elution process was monitored by spectrophotometry at 280 nm. Fractions eluted with ddH_2_O (2 ml/min × cm^2^) were ultrafiltrated and assayed for wilt-reducing activity as described below. Active fractions that eluted in a volume of Blue Dextran and contained high-molecular weight metabolites (HMWM) were combined, lyophilized and used in tests for *in vitro* toxicity, and further chromatographic separation followed by plant protection and defense response assays.

### Extraction of Fungal Biomass with Organic Solvents

Extracts with 70% aqueous methanol or a chloroform-methanol mixture (1:1) were obtained by shaking the powdered biomass in these solvents for 40–50 min at 4°C (Dorofeev et al., 2001). The solvents were removed from the extracts on a rotary evaporator. Residues were solubilized in ddH_2_O and freeze-dried.

### Ultrafiltration

Water-solubilized preparations of the extracts as well as fractions purified by gel filtration were passed through 5 kDa-cut-off membranes (Ultracel^^®^^- NMWCO 5K). Ultrafiltration was carried out using Amicon^^®^^ Ultra-15 Centrifugal Filter Units (Millipore). After centrifugation at 4000 × *g* for 15 min, permeates were collected. Retentates under the membrane were triply washed with ddH_2_O using a volume equal to the volume of the initial extract. The washed retentates were collected for analyses. To nullify an accompanying concentration effect of ultrafiltering and compare specific anti-wilt activity of HMWM and low-molecular weight compounds, the retentate volumes were adjusted to the volumes of the corresponding permeates. HMWM solutions were sterilized by passing them through a 0.22 μm Millipore filter prior to use.

### HMWM Testing for *in Vitro* Toxicity Against Pathogenic FOL Strains

Plant pathogenic FOL strains F37 and Fot3 were grown at 25°C for 7–10 days on potato dextrose agar (PDA). To prepare spore suspensions, 5 ml of sterile ddH_2_O (sddH_2_O) were added to the cultures and the culture surface were gently rubbed with an inoculation loop. The suspension was filtered through a sterile cotton to remove mycelium and centrifuged at 3000 *g* for 15 min. Conidia were re-suspended in and diluted with solutions of HMWM purified by gel filtration (0.5 mg of the lyophilized preparation per ml of ddH_2_O) to a final concentration of 10^6^conidia/ml. After hour-long incubation, 0.5 ml of the suspension was uniformly pipetted on glass slide covered with thin layer of 1% water agar. The slides were placed on moistened paper filters in Petri dishes. Germination was estimated after overnight incubation in the dark at 25°C by counting the number of germinated spores at 160× magnification. The average percentage germination relative to control (spores that germinated in sddH_2_O) was calculated for 300 spores in each treatment.

Lyophilized HMWM obtained using gel filtration were dissolved in a minimal volume of sddH_2_O and mixed with molten Czapek agar at 40°C to result in a final concentration of 0.5 g/ml, which was immediately poured into 6 cm diameter Petri dishes. Agar disks of F73 or Fot3 mycelia (5 mm in diameter) cut from the growing edge of PDA cultures were placed to the center of each plate with Czapek solidified agar. Three plates with the HMWM-augmented medium and three without HMWM (control) were inoculated with each strain. Colony diameters were measured after 6 days of cultivation in the dark at 25°C. Conidial germination and colony growth tests were repeated three times with newly obtained HMWM preparation in each independent experiment.

### Preparative Size-Exclusion Chromatography of HMWM

High-molecular weight metabolites isolated by gel filtration on Sephadex G-50 was lyophilized, dissolved in minimal volume of sddH_2_O, and guanidine-HCl was added for a complete solubilization of a water-undissolved residue. The solvent was centrifuged at 14000 × *g* for 10 min, the supernatant was separated by size-exclusion chromatography on a HiPrep26/60 Sephacryl S-400 HR column (GE Healthcare) equilibrated with sddH_2_O and held at 4°C. Fungal metabolites were eluted with freshly prepared ddH_2_O at a flow rate of 0.5 ml/min (10 min a 5-ml fraction) and detected at 280 nm. The collected 5-ml fractions were combined into seven fractions I–VII (see **Figure 3**). All samples except fraction VII (guanidine-HCl) were tested for ability to protect tomato seedlings. Absorption spectra of the tested fractions were recorded on a Hitachi U-3210 spectrophotometer (Hitachi, Tokyo, Japan) in the range of 180–320 nm. Fractions III, IV, and V with an UV-spectra typical for proteins and the absorption maximum at 214 nm were further analyzed by electrophoresis in 15% SDS-PAAG with or without mercaptoethanol (ME) followed by Coomassie staining.

### Plant Protection Assay

Four-week-old tomato plants (cv. Volgogradsky, 10–15 equal-sized seedlings per treatment) grown in pots with disinfected sand under control conditions (Shcherbakova et al., 2011) were gently removed from the substrate, and their roots were thoroughly washed with freshly prepared ddH_2_O, and then with sddH_2_O. Lyophilized samples that were tested for protective activity were dissolved in sddH_2_O. The seedlings were placed in these solutions to cover the roots but not the leaves. Control seedlings were immersed in sddH_2_O. After 2 days incubation at a room temperature under aseptic conditions, seedlings exposed to the tested samples and a portion of non-exposed seedlings were inoculated by a spore mixture of two pathogenic FOL strains F37 and Fot3 with final concentration of 10^6^ conidia/ml as described previously (Shcherbakova et al., 2011). After 2 days incubation with the pathogen, infected seedlings as well as non-inoculated control seedlings were re-planted and grown for 15 or 21 days. The wilt symptoms on inoculated tomatoes were assessed visually. Index of the disease for each individual plant was determined using a five-point rating scale (0 = healthy plants, 1 = 25%, 2 = 50%, 3 = 75% wilt severity, and 4 = dead plants). In some experiments, the disease severity (R, %) were also calculated by an appropriate formula (Shcherbakova et al., 2011).

### Protein Digestion Assay

Samples of the protein-containing fractions isolated by size-exclusion chromatography were treated with proteinase K (Promega, USA) following a protocol recommended by the manufacturer for native protein cleavage^1^. Briefly, the samples were dissolved in 50 mMTris-HCl buffer, pH 8.0; proteinase K was added into these solutions to a final concentration of 100 μg/ml; co-incubation with the enzyme was carried out at 37°C for 2 h; and the reaction was inhibited by 5 mM phenylmethylsulfonyl fluoride (PMSF). Biocontrol potential of the treated fractions was assessed as above except that control seedlings were exposed to not only water but also to 50 mM Tris-HCl amended with proteinase and PMSF at the same concentrations. Two independent experiments were carried out, each treatment was done in triplicate.

### Methods for Assessment of Elicitor Activity and Induced Plant Defense Responses

#### Extracellular Alkalinization Assay

Tomato cell (lines β and γ) were grown in Murashige and Skoog medium (4.4 g/liter) at pH 5.5, supplemented with sucrose (3%), myo-inositol (2.78 mM), α-naphthlaneacetic acid (5.4 μM) and *n*-benzyladenine (1.0 μM). Suspension cultures were maintained at a constant temperature (25°C) in the dark on an orbital shaker New Brunswick E-25R at 140 rpm and sub-cultured every 10 days by the addition of 8 ml of cell suspension to 60 ml of fresh complete medium. After 5–7 days, cells in the exponential growth phase were used in assays according to method developed by Felix et al. (1993). Portions of the suspension (8–10 ml) were transferred in 30 ml vials, and then equilibrated for 1–1.5 h at 25°C and 150 rpm on a shaker ELMI S-3.02 (ELMI Ltd.) before treatments were applied. Aliquots of 50–200 μl from the sterile ddH_2_O-dissolved lyophilized protein fraction V were added to cells, and changes in extracellular pH were monitored with time by using a universal pH-meter ZV-74 (ZIP, Russia) with an Orion semimicro pH electrode (Orion, USA) connected to a Cole Parmer recorder (USA).

#### Chitinase Activity Analysis

Root extracts of tomato seedlings were prepared as described by Pozo et al. (1998) except that 200 mM sodium phosphate buffer, pH 7.0 was used for extraction, and supernatants of centrifuged homogenates were concentrated by ultrafiltration. The chitin azure test described by Thompson et al. (2001) was applied to measure enzymatic activity in the extracts.

#### Tomato *PR1* Gene Expression Analysis

Seventy tomato seedlings were grown to the fourth true leaf stage at 27–28°C, 150 μE m^-2^ s^-1^ (16-h day period) and 60% relative humidity under aseptic conditions. Samples of second and third leaves of 10 seedlings were immediately frozen in liquid nitrogen (they are referred to as intact leaves). Thirty seedlings were placed in an aqueous solution of the freeze-dried protein-containing fraction V (200 μg/ml, 9 ml per each seedling) to cover their roots. For the control treatment, 30 seedlings were immersed in sddH_2_O (9 ml a plant). After 24 h of incubation under above conditions, samples of second and third leaves from 10 seedlings, which roots were treated with the fraction V, and from 10 control seedlings were harvested and immediately frozen in liquid nitrogen. Twenty remaining treated and 20 remaining control seedlings were inoculated with the pathogen by root soaking in a sterile spore suspension (see above). After a 48 h-inoculation period, leaf samples from 10 seedlings pre-exposed to the tested fraction as well as from 10 control those were collected and frozen in liquid nitrogen. All leaf samples were stored frozen at -70°C until RNA extraction. The remaining 20 pathogen-inoculated seedling (10 pre-treated with fraction V plus 10 water-treated ones) were transported to 50% Knop solution and kept for next 5 days to monitor development of wilt symptoms.

Expression of *PR-1* in the sampled leaf tissues was investigated by semi-quantitative RT-PCR (qRT-PCR). Total RNA were isolated with Trizol RNA prep 100 reagent (ISOGEN Co., Russia), treated with DNAse (DNAse I, 1 U/μliter, 1000 units, Fermentas, Lithuania) and reverse transcribed using a RevertAidTM, First Strand cDNA Synthesis Kit (Fermentas, Lithuania). Tomato *PR-1*-specific primers (forward, 5′ TCT TGC GGT TCA TAA C 3′) and - (reverse, 5′ CCA GTT GCC TAC AGG ATC ATA 3′), which flanked a common conserved sequence in genes encoding family of tomato PR-1 proteins, were synthesized by Syntol Co. (Russia) to amplify a *PR-1* fragment of 364 bp in size. A housekeeping β-tubulin gene used as a constitutively expressed endogenous control was amplified with the specific primers F-4056 and R-4057 (expected product of 610 bp in size). The reaction mixture consisted of 1xTaq buffer (with ammonium sulfate), 0.2 M of each dNTPs, 2 mM MgCl_2_, 0.5 pmol of each primer, 0.1 U Taq polymerases, and 50 ng of the first strand cDNA. The amplification program consisted of 35 cycles (20 s at 94°C, 20 s at 54°C, and 20 s at 72°C) and 30 cycles (40 s at 94°C, 40 s at 58°C, and 40 s at 72°C) for *PR-1* and β *-Tubulin* fragments, respectively. In both cases, the amplification was initiated with denaturation for 4 min at 94°C, and was terminated with a 10 min final step at 72°C. The PCR products were separated and visualized by electrophoresis in 1.5% agarose containing ethidium bromide (0.5 μg/ml), and results of PT-PCR were scanned and quantified by ImageJ software. The ratio of indications obtained for *PR-1* and β-*Tubulin* bands was calculated for each seedling, and the level of *PR-1*/β *-Tubulin* expression was averaged for each treatment. Two independent experiments were carried out.

### Methods Used for Identification of a CS-20 Elicitor Protein

#### HPLC of Proteins Isolated from HMWM

Protein-containing samples III, IV, and V, each sample individually, were pooled after the size-exclusion chromatography of five HMWM preparations independently isolated from CS-20 biomass and concentrated on a SpeedVac rotary concentrator to 2 ml. The concentrates were supplemented with guanidine-HCl and analyzed by reversed-phase HPLC (RF-HPLC) on an Aquapore C8 RP300 column (4.6 mm × 100 mm). Proteins were eluted with a 60-min linear acetonitrile gradient (10–60%) in 0.1% trifluoroacetic acid at a flow rate 0.7 ml/min and detected spectrophotometrically at 214 nm.

#### Protein Molecular Mass Determination and N-terminal Sequencing

The molecular weight of proteins discovered during RF-HPLC were evaluated by MALDI-TOF MS (Ultraflex MALDI-TOF-TOF mass spectrometer, Bruker Daltonics, Bremen, Germany). Calibration was performed using a ProteoMass peptide and protein MALDI-MS calibration kit (mass range 700–66 000 Da; Sigma–Aldrich). Molecular masses were determined in linear and reflector positive-ion mode. The samples prepared by the dried-droplet method with α-cyano-4-hydroxycinnamic acid (Sigma–Aldrich) as a matrix (10 mg/ml of 50% acetonitrile with 0.1% trifluoroacetic acid).

N-terminal amino acid sequences of the protein specific elicitor fraction V were determined by automated Edman degradation on a model 492 Procise sequencer (Applied Biosystems, Foster City, CA, USA).

#### Total RNA Isolation and cDNA Synthesis

Total RNA was extracted from freeze-dried fungal mycelium with TRIZOL reagent (Invitrogen, USA) according to the manufacturer’s protocol. The reverse transcription reaction was carried out in GNOM thermostat (DNA-technology, Russia) using RevertAid Premium First Strand cDNA Synthesis Kit (Thermo Scientific, USA) according to manufacturer’s instructions. The oligonucleotides containing (dT)_18_ with the adapters (dT)_18_AdLo and (dT)_18_AdLo2 (Ryazantsev et al., 2014) were used to initiate the first cDNA strand. Concentration of cDNA was estimated using NanoVue spectrophotometer (GE HealthCare, USA). Before adding to a PCR mix, cDNA samples were diluted to 50 ng/μl.

#### Rapid Amplification of cDNA Ends (3′ RACE)

3′ RACE was carried out in TERTSIK thermal cycler (DNA-technology, Russia) using the following reaction mix (total volume 35 μl): 3.5 μl 10x PCR buffer (750 Tris-HCl, PH 8.8; 200 MM ammonium sulfate; 0.1% Tween-20), 0.5 MM each of dNTP, 7 MM of primers, 2.5 units of Taq-polymerase and 3 μl of cDNA solution. The oligonucleotide pairs AdLo2-CS20F (CS20F: AARTGYGAYWSNGGNTGYTAYYTNAARGT) for the first round and AdLo-CS20F1 (CS20F1: GGNTGYTAYYTNAA RGTNTGYGAYTGYAGRAA; for AdLo and AdLo2 sequences see Ryazantsev et al., 2014) for the second round were applied (all oligonucleotides synthesized by Evrogen JSC, Russia). Properties of primers were estimated using the Oligo 6.71 program. The following optimized universal profile has been used for PCR amplification: 93°C–90 s; 93°C–20 s, 52°C–10 s; 72°C–10 s (35 cycles); 72°C–2 min. PCR products were separated and visualized by electrophoresis in 1% agarose gel run in 1x TAE buffer (40 mMTris; 20 mM ice-cold acetic acid; 1 mM EDTA) containing ethidium bromide (0.5 μg/ml). The GeneRuler^TM^ 1-kb DNA Ladder (Fermentas, Lithuania) was used to estimate the size of the target PCR product.

#### DNA Cloning and Sequencing

PCR products were purified with QIAEX II Gel Extraction Kit (Qiagen, Germany) followed by ligation of DNA fragments into pTZ57R/T vector and cloning according to the instructions of the InstA Clone PCR Cloning Kit (Fermentas, Lithuania). Transformation was carried out using *Escherichia coli* strain XL-1 Blue. Plasmid DNA was isolated with a GeneJet Plasmid Miniprep Kit (Fermentas, Lithuania). Sequence analysis was performed by Evrogen JSC using automatic sequencer ABI PRISM 3730 (Applied Biosystems, USA) with ABI PRISM BigDye^TM^ Terminator v. 3.1. reagents kit using a dideoxy chain termination method reaction (Sanger et al., 1977).

#### Phylogenetic Analysis

The predicted protein sequence of CS-20 was compared with sequences deposited in GenBank by BLAST^2^. A phylogenetic tree was constructed in MEGA5 software (Tamura et al., 2011) using maximum likelihood (ML) method and Jones-Taylor-Thornton (JTT) model (Jones et al., 1992). Bootstrap analysis with 1000 bootstrap replications was carried out to infer tree topology.

### Statistical Analysis

Quantitative data of the experiments were statistically analyzed with Microsoft software STATISTICA 6.0 (StatSoft Inc.). Means of different treatments, standard errors or standard deviations, and significant differences (*p* < 0.05) of means among treatments and controls were determined using a *t*-test for independent variables.

## Results

### Isolation of CS-20 Proteins Responsible for Protection of Tomato Plants from *Fusarium* Wilt Agent

**FIGURE 1:**
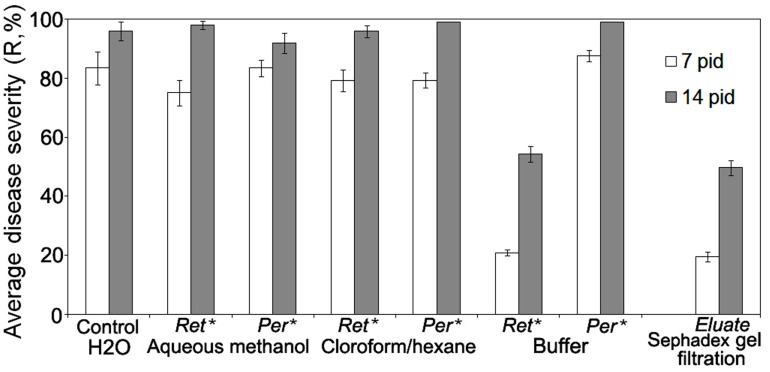
**Comparison of specific anti-wilt activity of different extracts from biomass of biocontrol *Fusarium oxysporum* strain CS-20 separated by ultrafiltration and gel filtration on Sephadex G-50.** ^∗^Ret.-retentate contains solutes greater than 5 kDa (are considered as high-molecular weight metabolites); Rer. -permeate contains low-molecular weight solutes. Water-solubilized preparations of freeze-dried extracts passed through 5 kDa-cut-off membranes were tested. Retentates and permeates were adjusted to equal volumes. Means of three independent experiments, with three replications per treatment in each ones, are presented. Bars represent SE. Activity in the permeates from samples extracted with the buffer were tested against H_2_O amended with salts, which remained in this permeate after isolation procedure used (see Materials and Methods, Ultrafiltration). Eluate means an active fraction that was eluted from Sephadex G-50 with double distilled water (see section ‘Materials and Methods’).

In order to find CS-20 metabolites conferring anti-wilt activity, we compared the disease development on seedlings pre-exposed to 70% water-methanol, chloroform/methanol or buffer extracts of fungal biomass, which had been subjected to ultrafiltration. Before inoculation by the *Fusarium* wilt pathogen, tomato seedling were incubated in water-solubilized preparations of the lyophilized extracts passed through 5 kDa-cut-off membranes. Seedling experiments on testing the compounds with NMWCO greater than 5 kDa (retentates) and permeates containing substances of lower molecular mass showed the target activity was associated with HMWM, which were extracted by the buffer (**Figure 1**).

Similar results were obtained when HMWM, which were purified by gel filtration (**Figure 1**) on a preparative Sephadex G-50 column, and then was tested for their ability to reduce wilt severity (**Table 1**). Wilt-reducing properties were detected in a high-molecular weight fraction that was eluted as a single peak in the column void volume, while other fractions did not influence wilt severity (data not shown). Fifteen-minute boiling fully inactivated the HMWM (**Table 1**).

**Table 1:** Reduction of wilt severity on tomato seedling pre-treated with high-molecular weight metabolites (HMWM) isolated from biocontrol *Fusarium oxysporum* strain CS-20.

Concentrations of HMWM eluted from Sephadex G-50, μg/ml	Days after root inoculation			
	15		21	
	Average wilt severity index	Wilt severity reduction respective to control, %	Average wilt severity index	Wilt severity reduction respective to control, %
200	0.7^c^	81	2.0^b^	49
50	2.0^b^	44	3.6^a^	8
10	2.5^b^	31	3.5^a^	10
5	3.5^a^	3	3.8^a^	3
0^∗^	3.6^a^	-	3.9^a^	-
200 + 100°C, 15 min	3.6^a^	0	3.8^a^	0

*^∗^No pre-incubation in HMWM prior to inoculation with the pathogen; seedlings were immersed in sterile double distilled water. Results represent means values calculated from means obtained in each of five experiments. Numbers within the columns denoted with different lower case letters differ from each other at P ≤ 0.05.*

**FIGURE 2:**
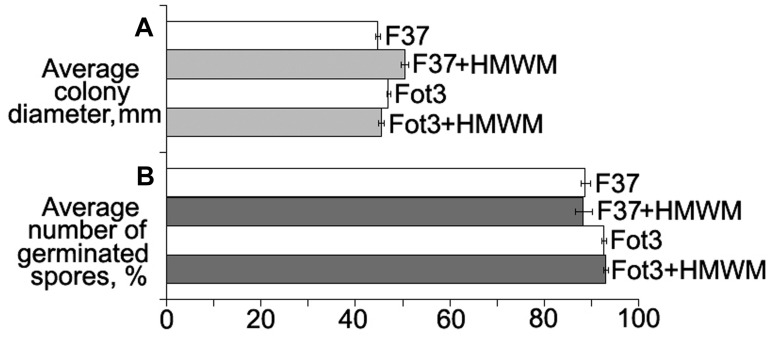
**Histograms showing the lack of direct toxic impact of high-molecular weight metabolites (HMWM) from *F. oxysporum* CS-20 toward pathogenic *F. oxysporum* f. sp. *lycopersici* strains (F37 and Fot3).** Mean values of three independent experiments, each of them comprised *in vitro* growth **(A)** and spore germination **(B)** tests, are presented. Bars represent SD.

Cultivation of two pathogenic strains F37 and Fot3 on Czapek agar supplemented with HMWM resulted in no retardation of the colony growth (**Figure 2A**). No inhibition of spore germination was found when spores of these strains were incubated in the tested metabolites (**Figure 2B**). Thus, protective effect of HMWM observed on tomato was not caused by their toxicity toward the pathogen.

**FIGURE 3:**
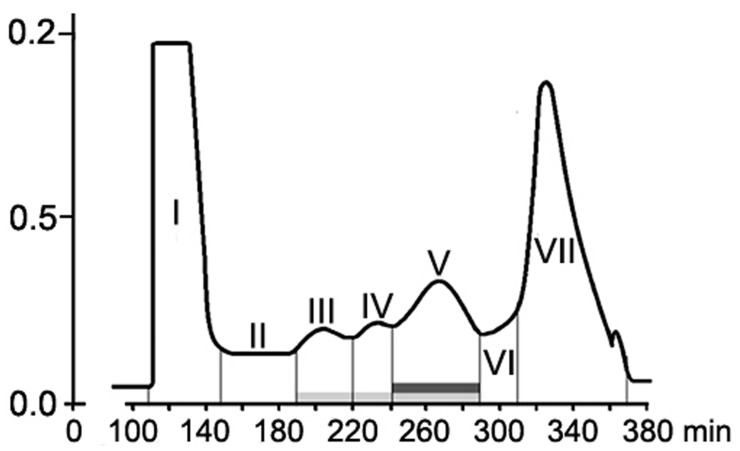
**Elution profile of *F. oxysporum* CS-20 high-molecular weight metabolites (HMWM) separated by size-exclusion chromatography on a column HiPrep26/60 Sephacryl S-400.** Protein constituents of HMWM are indicated by a light gray box; protein-containing fraction V possessing protective activity against *F. oxysporum* f. sp. *lycopersici* is marked with a dark gray box. Fraction VII represents guanidine-HCl, which was added to a HMWM solution before the chromatographic analysis.

**FIGURE 4:**
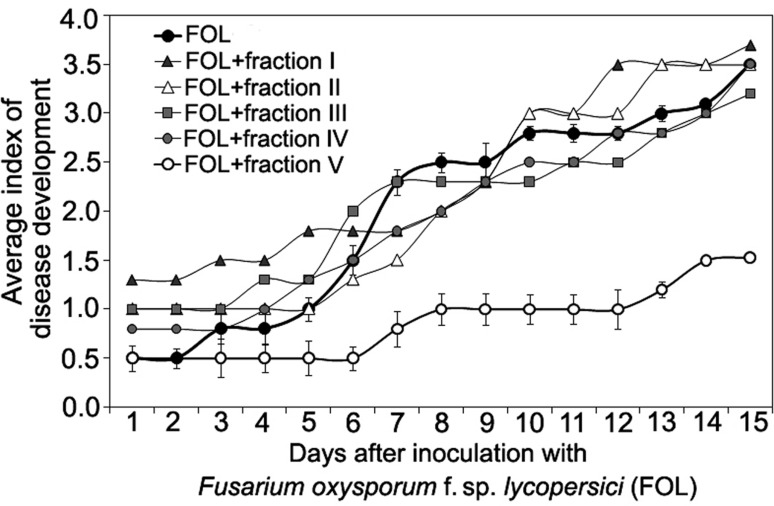
**Time course of *Fusarium* wilt on tomato seedlings treated with different fractions isolated by exclusion chromatography from high-molecular weight metabolites of a biocontrol *F. oxysporum* CS-20.** Prior to inoculation by pathogenic FOL strains (F37 + Fot3), roots of tomato seedlings were exposed to fractions I–V (see **Figure 3**) for 2 days. Control seedlings were exposed to sterile ddH_2_0. The fractions were sterilized by passing through a microporous filter. After 2 days of incubation under aseptic conditions, seedlings were planted, and disease symptoms were visually monitored. Mean values of the disease index from four independent experiments, five replications a treatment in each ones, are presented. Bars represent SD.

**FIGURE 5:**
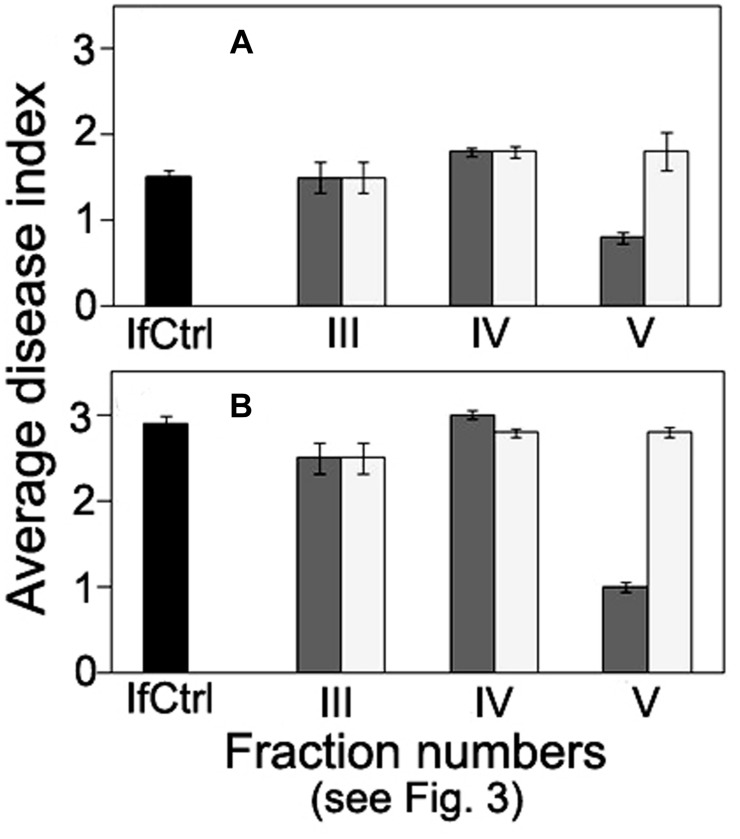
**Comparison of anti-wilt activity of the fractions obtained by exclusion chromatography of high-molecular weight metabolites from *F. oxysporum* strain CS-20.** Seedlings were inoculated by immersion in a suspension with a final concentration of 10^6^ spores/ml, containing spores of two pathogenic strains F37 and Fot3 (1:1). Disease symptoms were examined at 15 **(A)**, and 21 **(B)** days after inoculation of tomato seedlings, which roots were pre-exposed to intact (dark gray columns) or proteinase K-treated (light gray columns) fractions eluted as peaks III, IV, and V. Inoculated seedlings non-exposed to the fractions are referred to as infected control (IfCtrl, black columns). Histograms represent values of average disease index from two independent experiments of each treatment done in triplicate. Bars represent SD.

Size-exclusion chromatography on the column with Sephacryl S-400 showed the presence of two main (fractions I, VII), three minor (fractions III, IV, V) peaks, and two intermediate areas (fraction II, VI) in the elution profile of the HMWM (**Figure 3**). A plant assay to determine the possible role of the eluted portions in wilt reduction revealed no protective activity in fractions I, II (**Figure 4**). Fraction VI was contaminated with guanidine-HCl that was used for solubilization of the frozen-dried HMWM preparation prior to the chromatographic analysis. This fraction was phytotoxic and was excluded from the further studies. The fractions eluted in the range of peaks from III to V were not phytotoxic and contained colorless opalescent substances, which absorbed in the range of 180–230 nm with a maximal absorbance level near 214 nm (data not shown). These spectral characteristics suggested that fractions III–V contained proteins. Indeed, a number of bands covering a relatively wide range of molecular weights lower than 66 kDa were revealed in these fractions by SDS-PAGE (patterns not shown). In fraction V, the number of bands with a higher electrophoretic mobility increased after its separation in a ME-containing gel, suggesting the presence of proteins with inter-chain S–S bonds. Among the three protein-containing fractions, anti-wilt activity was found only in fraction V (**Figure 4**). Incubation of seedling roots in fraction V before plant inoculation with the pathogen significantly reduced the disease index resulting in a sustainable plant protection throughout the observation time, while other protein fractions (III and IV) did not prevent or delay wilt development (**Figure 4**). Hydrolysis of the active fraction V with proteinase K nullified its protective effect, but did not make any impact on wilt development if seedlings were pretreated with other protein fractions (**Figure 5**). The loss of protection properties after enzymatic proteolysis demonstrated that the activity of HMWM against *Fusarium* wilt agent was determined by their proteinaceous constituent. Moreover, reduction in disease severity on tomato seedlings due to the pre-inoculation exposure of roots to the fraction V together with above *in vitro* experiments on cultivation pathogenic FOL strains at the presence of HMWM denoted plant-mediated mode of action of the isolated proteins, such as an induction of plant defense responses involved in induced resistance.

### Defense Responses Induced in Tomato Plants by Protective Proteins from CS-20

To establish whether the proteins protecting tomatoes acted as disease resistance elicitors, their ability to induce plant defense responses involved in local and systemic resistance against various causative agents including pathogenic FOL was investigated.

#### Induction of Extracellular Alkalinization

**FIGURE 6:**
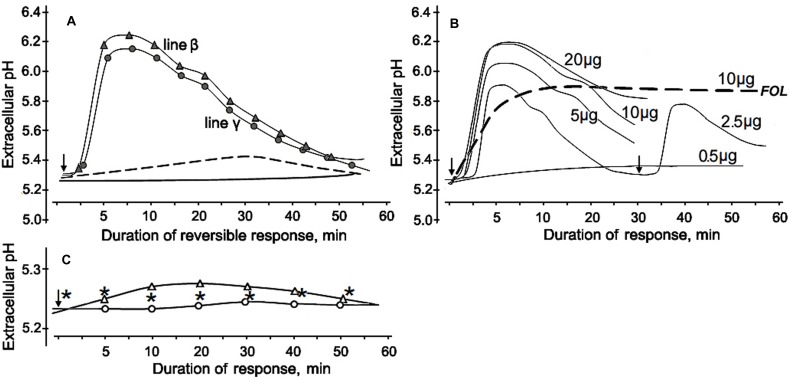
**Alkalinization of the incubation medium by cultured tomato cells in response to CS-20 proteins contained in fraction V, which reduced *Fusarium* wilt severity in tomato seedlings. (A)** Profiles of extracellular pH change in suspensions of two cell lines after addition of the lyophilized fraction V to a final concentration of 10 μg/ml. Cell response (line β) to the fraction pretreated with proteinase K shown by dotted line. The solid line shows extracellular pH of non-treated cells. **(B)** Dose-rate effect of the fraction V isolated from CS-20 on tomato cell line γ. Numerals near curves show final concentrations (μg per ml) of the lyophilized fraction V in the tomato cell suspension. Re-stimulation of the reversible response is exemplified for a concentration of 2.5 μg/ml. Dotted line illustrates the irreversibility of alkalinization response to preparation from the pathogenic FOL (strain F37), obtained by the same procedures that were applied to isolate fraction V from CS-20. **(C)** The extracellular pH values after addition of inactive fractions III (white circles) or IV (white triangles) at a concentration of 10 μg/ml. Asterisks show extracellular pH of non-treated cells. Arrows indicated starting point of treatments. The representative data are out of one of three experiments with the protein fraction V samples independently isolated from CS-20 culture.

The first approach to obtaining data on putative eliciting properties of the tested proteins was investigation of their alkalinization-inducing activity. The protein-containing fraction V that reduced wilt on tomato seedlings was also effective in elicitation of a reversible change of extracellular pH in cultured tomato cells. A rapid alkalinization of the extracellular medium was observed in response to addition of the lyophilized preparation of this fraction into the cell suspensions of two tomato lines used in our experiment. Extracellular pH started to increase from the initial level after a 2.5–3 min lag phase and reached a maximum to 5–10 min followed by a gradual subsidence over the next 50–50 min (**Figure 6A**). The fraction V induced a reversible response of similar profile with close values of ΔpH in both cell lines. The cells did not react to ddH_2_O passed through column with Sephacryl S-400 after passing the extraction buffer, and addition of the tested fraction in the cell-free incubation medium up to a final concentration of 100 μg/ml did not influence extracellular pH, indicating that there was no artifact alkalinization due to the eluent or the protein solution itself. The increased pH level and the cell, response duration positively correlated with amount of the added fraction, however, the dose-response effect was not linear (**Figure 6B**). The tested fraction at 0.5 μg/ml did not induce any significant reaction, while the highest ΔpH averaged 0.84, if the fraction was added to a concentration of 10 μg/ml. Further increase of the concentration produced no elevation of pH although cell response was still reversible. Fractions III and IV, which were not able to mitigate wilt symptoms, did not elicit any significant change in extracellular pH (**Figure 6C**). Cells treated with proteins containing in the fraction V remained viable and could be re-stimulated at repeated contact with it, herewith the level of the medium alkalinization during the second response was almost as high as upon the first stimulation (**Figure 6B**). In contrast, a preparation obtained from the pathogenic FOL by the same isolation procedures that were used for purification of CS-20 proteins initiated a gradual slow growth of extracellular pH without any visible lag phase, produced irreversible response (**Figure 6B**), led to cell death and browning of the suspension. The cleavage products of the fraction V pretreated with proteinase K were almost completely inactive as alkalinization inducers. In this case, difference between control and the treatment did not go beyond 0.1 pH unit even if the inactivated preparation was added to a concentration 10-fold exceeded the effective concentration of the native fraction (**Figure 6A**). These results indicate proteins contained in fraction V that we isolated from CS-20 strain were able to elicit the ion exchange response in tomato cells, a well-known early non-specific defense reaction governing plant–pathogen relationships.

#### Chitinase Activity

**FIGURE 7:**
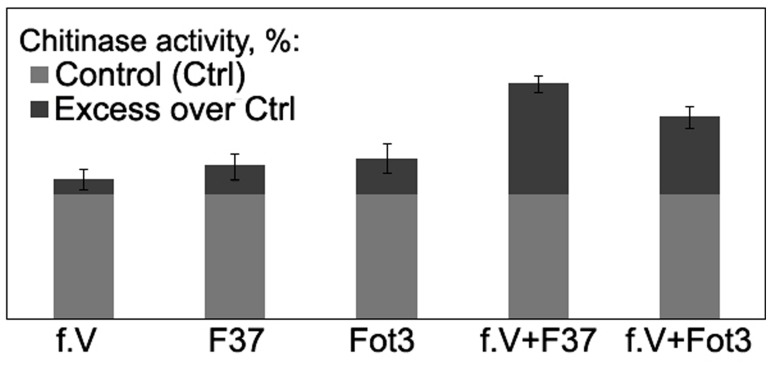
**Histograms showing percentage chitinase activity relative to control in root extracts obtained from tomato seedlings exposed to protein fraction V (f.V), pathogenic *F. oxysporum* f. sp. *lycopersici* strains F37 or Fot3 and in similar extracts from F37- or Fot3-inoculated seedlings, which were exposed to f.V prior to contact with pathogenic strains.** The level of chitinase activity in extracts from roots of non-treated and non-inoculated seedlings are referred to as control (Ctrl) and taken as 100%. Presented data are means from one of two experiments, both of them showed similar results. Bars represent SE.

To gain more information about mode of action of the isolated proteins, we analyzed activity of chitinase, one of the PR-proteins closely associated with interrelationships of several plant species with *Fusarium* wilt agents (e.g., Benhamou et al., 1990; Cachinero et al., 2002) including tomato-FOL interactions, and biocontrol of *Fusarium* wilt of tomato (Recorbet et al., 1998; Fravel et al., 2003b; Aime et al., 2013). In our experiments, inoculation of untreated tomato seedlings by the pathogen alone as well as a 3-day incubation in protein fraction V alone slightly enhanced chitinase activity in root extracts, while pre-exposure of tomato roots in the fraction V resulted in 1.6- or 2.3-fold stimulation of the enzyme in response to a subsequent contact with the pathogenic FOL strains Fot3 or F37, respectively (**Figure 7**).

#### *PR-1* Expression

**FIGURE 8:**
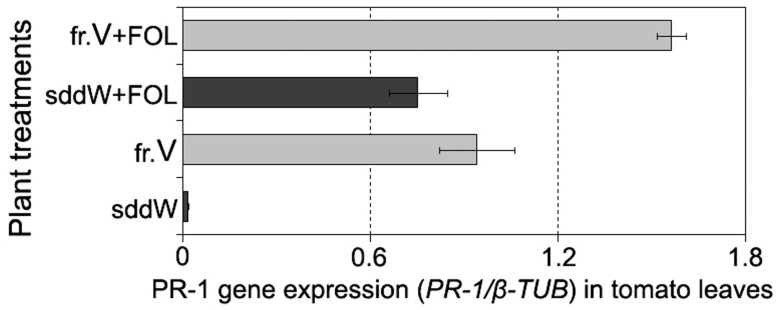
**Histogram showing an enhanced *PR-1* expression in leaves of tomato seedlings in response to root treatment with the protein fraction V isolated from CS-20 biomass (fr.V) and after subsequent inoculation with the pathogen (fr.V + FOL) compared to corresponding control treatments.** Seedling roots were exposed to fr.V for 24 h. Roots of control seedlings were inoculated with FOL after 24 h of incubation in sterile double distilled water (sddW).

Analysis of PR-1 expression in tomato leaves exposed to fraction V showed this defense gene systemically responded in plants to the treatment with those CS-20 proteins that mitigated wilt symptoms and manifested elicitor properties in cultured cells. The target product of qPT-PCR of 364 bp in size was found in leaves from seedlings, roots of which were immersed in the elicitor fraction V for 24 h. Over the same time period, no accumulation of *PR-1* was detected in leaves sampled from water-treated (control) seedlings compared to intact leaves. Root exposure to fraction V proteins activated the PR-1 protein gene, but gene expression was higher in tomato leaves of the seedlings, whose roots were exposed to the fraction V prior to the pathogen attack (**Figure 8**).

While both treated and control plants showed wilt symptoms at 7 days after inoculation, the severity and incidence of leaf yellowing and wilting were significantly less in protein-pretreated seedlings compared to control (wilt severity indices averaged 0.5 and 1.2, respectively, *p* = 0.02). These data demonstrate the fraction V induces a systemic resistance in tomato to FOL and suggest that one of mode of the protein action may be elicitation of plant response pathways involving PR-1.

### Determination of Amino Acid and Nucleotide Sequences of a Putative Elicitor Protein from CS-20

**FIGURE 9:**
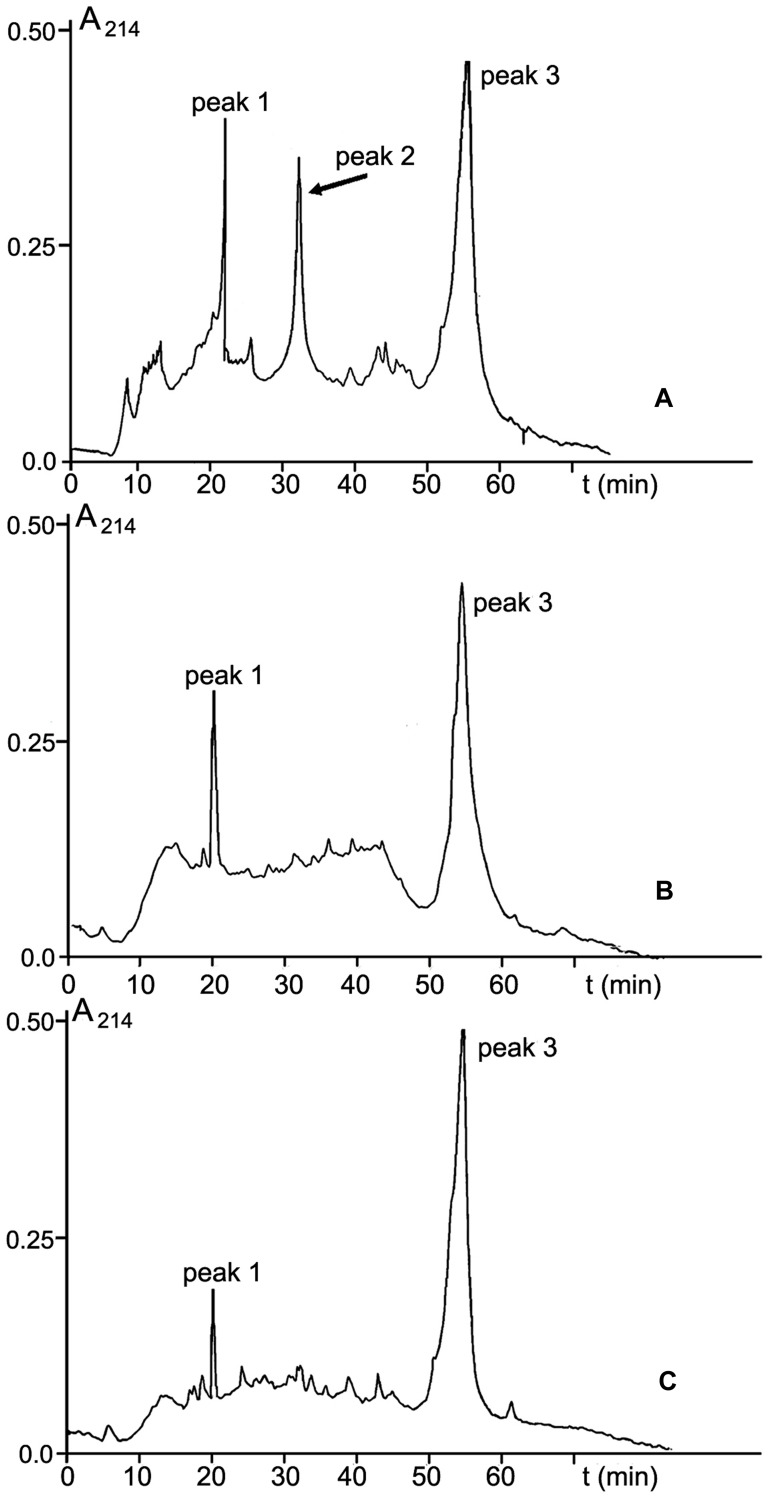
**RP-HPLC on an Aquapore C8 RP300 (4.6 mm × 100 mm) column of fractions V (A), III (B), and IV (C) obtained by SE-chromatography on Sephacryl S400 (see **Figure 3**).** For experimental details, see section ‘Materials and Methods.’ The 10.033-Da protein is shown by an arrow.

Among three protein-containing fractions isolated from CS-20 biomass, only fraction V elicited local and systemic plant defense responses that reduced wilt symptoms on tomato exposed to the *Fusarium* wilt pathogen. In this context, we presumed that elicitor properties of this fraction, and consequently, its ability to protect tomato against FOL were determined by some proteins, which were absent in two other protein fractions. To confirm this hypothesis, fraction V from size-exclusion chromatography was subjected to RP-HPLC. Inactive fractions III and IV were separated in parallel (**Figure 9**). Three main peaks with retention time (RT) 20, 32, and 55 min were present in RP-HPLC chromatogram of the elicitor fraction (**Figure 9A**). Two of them, peaks 1 and 3 (RT 20 and 55 min, respectively), were also found in the elution profiles of the inactive fractions III and IV (**Figures 9B,C**). MALDI-TOF MS showed these peaks were formed by polypeptides of similar molecular masses in all tested fractions (peak 1: 20000 Da; peak 3: 28410, 38971, 53917 19214, 32534 Da). The occurrence of these identical polypeptides in active and inactive fractions makes it highly improbable that elicitor and anti-wilt activity of fraction V was associated with these common constituents. By contrast, a polypeptide with molecular mass of 10033 Da eluted at 32 min (**Figure 9A**, peak 2) was specific to the fraction V, and was missing from the inactive fractions. Collectively, these findings suggest the 10033-Da protein is a likely candidate for elicitation of plant defense responses resulting in significant reduction of wilt symptoms on tomato seedlings after pre-treatment with CS-20 proteins presented in the fraction V.

**FIGURE 10:**
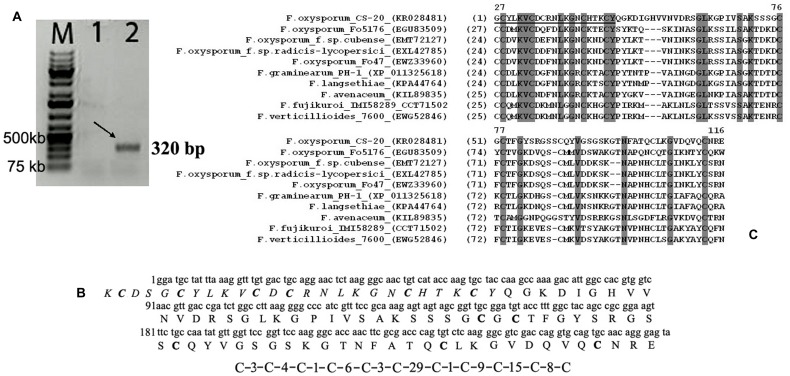
**A representative result of electrophoretic analysis of PCR-products amplified by two-step 3′ RACE (A); nucleotide and deduced amino acid sequences of the elicitor protein from *F. oxysporum* CS-20 – CS20EP (B); as well as the alignment of predicted CS20EP amino acid sequence with related sequences deposited in GenBank (C). (A)** The arrow indicates the target PCR-product of an estimated size (320 bp) that was amplified after the second 3′RACE round with AdLo-CS20F1 primer pair at different annealing temperatures. Lane 1, 60°C; line 2, 52°C; M, GeneRuler^TM^ 1 kb DNA ladder plus. **(B)** The predicted amino acid sequence of the CS-20 elicitor protein (CS20EP) is shown below the nucleotide sequence (GenBank accession number KR028481). The fifty-five amino acid residues corresponding to those determined by N-terminal sequencing of the protein are marked in italic. Cysteine residues in the amino acid sequence are marked in bold. The cysteine motif is presented under the sequence. **(C)** Identical amino acid residues in sequences are highlighted by dark-gray boxes. The CS20EP N-terminal sequence determined by Edman’s degradation is underlined.

The N-terminal sequence of the 10033-Da protein (25 amino acid residues: ^1^KCDSGCYLKVCDCRNLKGNCHTKCY^25^) was determined by automated Edman degradation. The sequenced region was found to be enriched in basic and cysteine amino acid residues. To determine the complete amino acid sequence of the 10033-Da protein, the protein-encoding cDNA was generated by two-step 3′-RACE. Because we failed to obtain a homogenous amplification product after one-step 3′-RACE, the second 3′-RACE round was carried with use of AdLo-CS20F1 inner primer pair, where CS20F1 was complementary to the cDNA fragment encoding the N-terminal sequence starting from the fifth amino acid residue. The deduced amino acid sequence of the protein, therefore, started from the fifth amino acid residue. Electrophoresis of the products after two-step 3′-RACE demonstrated the presence of a single amplicon of 320 bp in size (**Figure 10A**). This product was cloned and sequenced. The obtained nucleotide sequence (GenBank accession number KR028481) was translated into an amino acid sequence containing 90 residues, while the four first amino acids (^1^KCDS^4^) in the N-terminal fragment of the polypeptide were determined on the base of Edman chemistry. Thus, the complete primary structure of the CS-20 elicitor protein (CS20EP) was reconstructed from direct Edman and cDNA sequencing (**Figure 10B**). The calculated molecular mass of the deduced polypeptide of 94 amino acid residues is 9940.15 Da. The discrepancy between the calculated and measured molecular mass values suggests post-translational modifications of the molecule. The identified protein is basic, and its pI is 9.87. Uneven distribution of basic residues should be especially noted, the N-terminal region of the polypeptide being particularly enriched. CS20EP is also cysteine-rich. It contains a cysteine motif with eleven cysteine residues in the molecule, six of them were located in N-terminal region. Hydrophobic amino acid residues comprise 23.43%.

BLAST search in the NCBI database revealed no high homology to fungal, bacterial or plant proteins, including antifungal proteins and peptides, suggesting the protein is new, although it displays the similarity with other predicted polypeptide sequences (especially within their N-terminal regions and cysteine motifs) from *F. graminearum*, *F. pseudograminearum*, *F. langsethiae*, *F. avenaceum*, *F. oxysporum*, *F. verticillioides*, and *F. fujikuroi*. The highest similarity (47.8%) with a hypothetical protein FGSG_10784 from *F. graminearum* strain PH-1 was found (**Figure 10C**).

**FIGURE 11:**
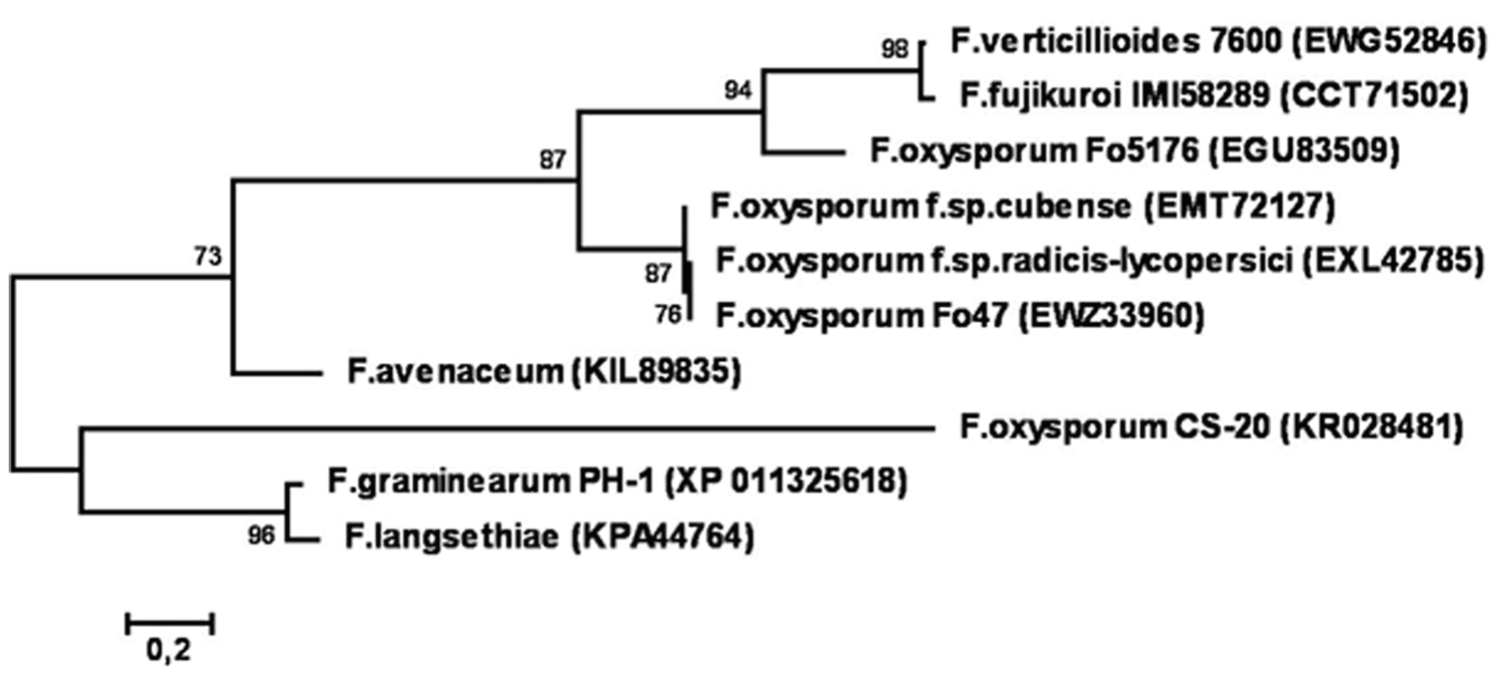
**Phylogenetic tree reconstructed for sequences of CS20EP and its homologs from different *Fusarium* species.** Only bootstrap values greater than 70% are indicated.

Phylogenetic reconstruction based on amino acid sequences of CS-20 and similar proteins deposited in GenBank (**Figure 11**) demonstrated that CS-20 represents a separate branch located between *F. graminearum*/*F. langsethiae* cluster (bootstrap support 96%) and *F.avenaceum*/*F.oxysporum*/*F.fujikuroi*/*F.verticillioides* cluster (bootstrap support 73%).

## Discussion

In filamentous fungi, as well as in bacteria and oomycetes, metabolites belonging to various chemical families are characterized as general or specific elicitors of plant defense responses, and some of most well characterized elicitors are proteins produced by fungi (Bailey, 1995; Takenaka et al., 2003; Hanson and Howell, 2004; Garcia-Brugger et al., 2006; Mishura et al., 2009; Zhang et al., 2010; Peng et al., 2011; Chen et al., 2012; Wang et al., 2012; Thakur and Sohal, 2013; Oomea et al., 2014).

In our study, the search for CS-20 metabolites modulating defense responses of tomato resulted in identification of a new putative elicitor belonging to a family of small cysteine-rich proteins. Proteins of this type are often involved in relationships among plants and microorganisms, as well as in interactions between plants and biocontrol fungi. Small cysteine-rich proteins are well documented in contributing to biocontrol by *Trichoderma* sp. For instance, Djonović et al. (2006) isolated, identified and characterized a small protein1 (Sm1) that was secreted by *Trichoderma virens* strain Gv29-8 in culture filtrates. Sm1 elicited local and systemic defense responses in cotton cotyledons and protected them from colonization by *Colletotrichum* sp., a foliar pathogen causing anthracnose. Analysis of the deduced amino acid sequence of Sm1 revealed the hydrophobic character of this protein, the presence of four cysteine residues and several sites for possible post-translational modifications (Djonović et al., 2006). The predicted molecular mass of the mature protein was determined to be 12.55 kDa (pI 5.78). Elicitor activity was also found in two other hydrophobins from a group of low-molecular weight, 4-cysteine-containing fungal proteins, EPL1 (12.6 kDa, pI 5.5–5.7) and EPLT4 (homolog of EPL1), produced by *T. atroviride* and *T. asperellum*, respectively (Seidl et al., 2006; Wang et al., 2013). Recently, a novel hydrophobin and its encoding gene were obtained from *T. longibrachiatum* strain MK1 (Ruocco et al., 2015). It was termed HYTLO1 (originally designated as protein HYTRA1 isolated from *T. harzianum* T22). Along with direct inhibition of some microbes and plant growth promotion activity, HYTLO1 is a strong elicitor of plant defense responses. Infiltration of HYTLO1/HYTRA1 in tomato leaves resulted in development of local and systemic resistance against *Botrytis cinerea* via a hypersensitive reaction, a generation of an oxidative burst in plant cells, and enhanced transcription or activity of PR-proteins. HYTLO1 was predicted to be 7.2 kDa cysteine-rich protein with eight cysteine residues arranged in a strictly conserved motif (Ruocco et al., 2015).

During colonization of plant tissues, plant pathogenic fungi secrete an arsenal of enzymes and effector proteins (Doehlemann and Hemetsberger, 2013), which are considered pathogenicity factors (Houterman et al., 2008; Dodds et al., 2009). At the same time, these enzymes can elicit plant defense responses (Rotblat et al., 2002), and many proteinaceous effectors are recognized by plants as avirulence gene products inducing host resistance (Rooney et al., 2005; Lorang et al., 2007; Dodds et al., 2009; Stergiopoulos and de Wit, 2009; Oliva et al., 2010). Small cysteine-rich proteins are well known as fungal effectors. The resistance of tomato against *Cladosporium fulvum* is highly correlated with secretion by the fungus of low-molecular weight cysteine-rich proteins, which are recognized by corresponding resistance genes in the plant (De Wit and Joosten, 1999; De Wit et al., 2002).

Interrelationships between pathogenic FOL and tomato are also regulated by small effector proteins enriched with cysteine. One such protein, named SIX1, was the first avirulence factor reported for FOL and other root-invading pathogens (Rep et al., 2004). After maturation of a 32 kDa a precursor, a central part of SIX1, the 12 kDa protein containing six cysteine residues is secreted in xylem sap of infected plants and induces defense responses in tomato lines with the *I-3* resistance gene (Rep et al., 2004). At least eleven small cysteine-rich proteins of the SIX type were identified in *F. oxysporum*, and some of them were shown to play a role of avirulence factors required for *I* gene-dependent tomato resistance against pathogenic FOL (Rep et al., 2004; Houterman et al., 2007, 2009; Takken and Rep, 2010).

In many plant-microbe interactions, the presence of an elicitor in the secretome of a biocontrol fungus is regarded as being crucial for induction of defense responses and protection. Since in this study we isolated CS20EP from fungal biomass and did not analyze the culture liquid, we cannot determine whether the isolated protein is a secreted elicitor, although bioinformatics analysis of its sequence using ESLPred2 method (Garg and Raghava, 2008) suggested that CS20EP might be an extracellular protein. Another secretion criteria could be the presence of a signal peptide and no predicted transmembrane helices in CS20EP precursor by both neural network and HMM prediction methods (Stassen et al., 2012). As 3′-RACE were used for sequencing of cDNA fragment for mature protein, we did not obtain the full-length cDNA and did not examine as yet whether CS20EP precursor contains a signal peptide. However, all the similar and phylogenetically related proteins from the analyzed *Fusarium* strains, including PH-1 and the biocontrol strain Fo47 (**Figures 10** and **11**), contain a conserved signal peptide of 23 amino acid residues (see Supplementary Figure S1) and hence, are secreted proteins. In addition, some molecular properties of CS20EP are similar to those of known secreted cysteine-rich effectors (e.g., Bolton et al., 2008; Stergiopoulos et al., 2012; Ökmen et al., 2013). As was mentioned above, many of them are less than 150 amino acids long, hydrophobic polypeptides with molecular mass ranging from several to twenty-thirty kDa and a cysteine content higher than 3%. Intramolecular disulfide bridges are supposed to stabilize protein tertiary structure in the harsh environment such as the plant apoplast (Djonović et al., 2006; Stergiopoulos and de Wit, 2009; Takken and Rep, 2010). CS20EP was also found to be small, hydrophobic, extremely enriched in cysteine residues (11%) with cysteine motif almost the same as in the related proteins (**Figure 10C**). While further research is needed to determine whether this protein is secreted, the properties discovered so far together with bioinformatics data suggest that it is probably secreted by CS-20.

Based on results reported here, it is possible to speculate that the plant-mediated mode of action of biocontrol by *F. oxysporum* strain CS-20 could be partially underlain by its ability to synthesize elicitors of systemic resistance, and CS20EP could be one of them. We identified the primary structure of CS20EP and determined nucleotide sequence encoding this protein. As far as we are aware, CS20EP is the first small cysteine-rich protein from biocontrol *F. oxysporum* strains putatively acting as an elicitor of defense responses in tomato against pathogenic FOL. In contrast to hydrophobins and cysteine-rich proteins from FOL, it is a basic protein. Interestingly, no homolog of *SIX1* was present in the genome sequence of *F. graminearum* (Rep et al., 2004), while the instance of relatively high similarity was revealed just between CS20EP and a nucleotide sequence encoding a hypothetical protein FGSG_10784 from *F. graminearum.*

Since the fungus produced CS20EP in amounts insufficient for biological assays, we tested it in the composition of the active fraction. Thus, currently only indirect evidence of the protective activity of this protein is available. First, MALDI-TOF MS analysis allowed us to identify several components in active and inactive fractions, with the 10-kDa polypeptide being present exclusively in the active fraction displaying elicitation of defense responses, while other components occurred both in active and inactive fractions, and we did not observe any elicitation of host defense response or wilt severity-reducing activity in these CS20EP-free fractions in our experiments. Secondly, the addition of fractions III and IV to fraction V did not increase its anti-wilt effect (data not shown). Thirdly, the UV spectrum of the pathogenic FOL strain F37 elutate portion, which corresponded to fraction V from CS-20 but did not reduce wilt on tomato seedlings, was atypical for proteins, and no polypeptides were detected in its composition by MS. Collectively, these findings support the hypothesis that CS20EP likely contributes to or is responsible for the elicitor and protective activities of fraction V. Since some elicitors have been reported to be perceived by cells even in trace amounts (Mueller et al., 2012), additional research is needed to determine if some other fungal products are involved in the observed protective activity and elicitation of defense responses.

Determination of the CS20EP-encoding nucleotide sequence reported here will facilitate the use of other approaches, such as development of super-producers of this elicitor or heterologous expression in order to obtain the protein at preparative amounts necessary for plant treatment and other biological assays. Experiments with individual CS20EP are also needed to investigate modes of action and elucidate role of this protein in the producer strain as well as its involvement in CS-20 biocontrol effect. Production of mutants with knockouts for CS20EP gene or fungal clones transformed for the protein will also be helpful for study its function and confirming its elicitor properties in our further studies. This research on CS20EP identification will enable a more targeted implementation of above approaches.

Our research showed pre-inoculation treatment of plants with the CS20EP-containing fraction resulted in a local increase of chitinase activity and systemic up-regulation of *PR-1* expression in response to the pathogen challenge. These mechanisms, along with evident activation and priming of other defense genes, were previously demonstrated for endophytic *F. oxysporum* strains V5w2 (Paparu et al., 2007) and Fo47 (Veloso and Diaz, 2012; Aime et al., 2013), which protect tomato and pepper (Fo47) as well as banana (V5w2) by induction of the resistance to FOL, *Verticillium dahliae, Phytophthora capsici* (Fo47) and nematode *Rodopholus similis* (V5w2). It is noteworthy that among three defense genes up-regulated by Fo47 in roots of pepper plants, only *PR-1* expression was also enhanced systemically (Veloso and Diaz, 2012). This makes it possible to surmise that similar responses are involved in the protective effect of CS-20, where CS20EP is one of the ‘molecular tools’ triggering plant defense mechanisms resulting in systemic acquired resistance. Elicitation of reversible ion exchange in tomato cells by fraction V allows making an assumption that CS20EP initiated early defense events through plant receptor-mediated recognition. Given the ability of strain CS-20 to produce CS20EP, which stimulates *PR-1* expression in tomato leaves, and the fact that the protein PR-1 is a marker of SA-dependent defense pathways, generally working most effectively against biotrophs, future research is planned to determine the protection potential of this biocontrol agent and also CS20EP, as an elicitor of systemic resistance, against foliar biotrophic pathogens of tomato.

Mention of a trademark or proprietary product does not constitute a guarantee or warranty of the product by the United States Department of Agriculture and does not imply its approval to the exclusion of other products that may be suitable.

## Conflict of Interest Statement

The authors declare that the research was conducted in the absence of any commercial or financial relationships that could be construed as a potential conflict of interest.

## Footnotes

1 http://worldwide.promega.com/resources/protocols/product-information-sheets/n/proteinase-k-protocol

2 http://blast.ncbi.nlm.nih.gov/Blast.cgi

## References

[B1] Aime S. Alabouvette C. Steinberg C. Olivain C. (2013). The endophytic strain *Fusarium oxysporum* Fo47: a good candidate for priming the defense responses in tomato roots. *MPMI* 26 918–926. 10.1094/MPMI-12-12-0290-R 23617416

[B2] Alabouvette C. Olivain C. Migheli Q. Steinberg C. (2009). Microbiological control of soil-borne phytopathogenic fungi with special emphasis on wilt-inducing *Fusarium oxysporum*. *New Phytol.* 184 529–544. 10.1111/j.1469-8137.2009.03014.x 19761494

[B3] Anitha A. Rabeeth M. (2009). Control of Fusarium wilt of tomato by bioformulation of *Streptomyces griseus* in green house condition. *Afr. J. Basic Appl. Sci.* 1 9–14.

[B4] Bailey B. A. (1995). Purification of a protein from culture filtrates of *Fusarium oxysporum* that induces ethylene and necrosis in leaves of *Erythroxylum coca*. *Phytopathology.* 85 1250–1255. 10.1094/Phyto-85-1250

[B5] Benhamou N. Charest P. M. Jarvis W. R. (1989). “Biology and host-parasite relationships of *Fusarium oxysporum*,” in *Vascular Wilt Diseases of Plants*, eds Tjamos E. Beckman C. (Berlin: Springer-Verlag), 95–105.

[B6] Benhamou N. Joosten M. H. A. J. De Wit P. J. G. M. (1990). Subcellular localization of chitinase and of its potential substrate in tomato root tissues infected by *Fusarium oxysporum* f. sp. radicis-lycopersici. *Plant Physiol.* 92 1108–1120. 10.1104/pp.92.4.1108 16667378 PMC1062423

[B7] Bolton M. D. van Esse H. P. Vossen J. H. de Jonge R. Stergiopoulos I. Stulemeijer I. J. E. (2008). The novel *Cladosporium fulvum* lysine motif effector Ecp6 is a virulence factor with orthologues in other fungal species. *Mol. Microbiol.* 69 119–136. 10.1111/j.1365-2958.2008.06270.x 18452583

[B8] Cachinero J. M. Hervas A. Jimenez-Diaz R. M. Tena M. (2002). Plant defense reactions against fusarium wilt in chickpea induced by incompatible race 0 of *Fusarium oxysporum* f. sp. ciceris and nonhost isolates of *F. oxysporum*. *Plant Pathol.* 51 765–776. 10.1046/j.1365-3059.2002.00760.x

[B9] Chen M. Zeng H. Qiu D. Guo L. Yang X. (2012). Purification and characterization of a novel hypersensitive response-inducing elicitor from *Magnaporthe oryzae* that triggers defense response in rice. *PLoS ONE* 7:e37654 10.1371/journal.pone.0037654 PMC3356297 22624059

[B10] De Wit P. J. Brandwagt B. F. van den Burg H. A. Cai X. van der Hoorn R. A. de Jong C. F. (2002). The molecular basis of co-evolution between *Cladosporium fulvum* and tomato. *Antonie Van Leeuwenhoek* 81 409–412. 10.1023/A:1020553120889 12448739

[B11] De Wit P. Joosten M. H. (1999). Avirulence and resistance genes in the *Cladosporium fulvum*-tomato interaction. *Curr. Opin. Microbiol.* 4 368–373. 10.1016/S1369-5274(99)80065-4 10458978

[B12] Djonović S. Pozo M. J. Dangott L. J. Howell C. R. Kenerley C. M. (2006). Sm1, a proteinaceous elicitor secreted by the biocontrol fungus *Trichoderma virens* induces plant defense responses and systemic resistance. *MPMI* 19 838–853. 10.1094/MPMI-19-0838 16903350

[B13] Dodds P. N. Rafiqi M. Gan P. H. P. Hardham A. R. Jones D. A. Ellis J. G. (2009). Effectors of biotrophic fungi and oomycetes: pathogenicity factors and triggers of host resistance. *New Phytol.* 183 993–999. 10.1111/j.1469-8137.2009.02922.x 19558422

[B14] Doehlemann G. Hemetsberger C. (2013). Apoplastic immunity and its suppression by filamentous plant pathogens. *New Phytol.* 198 1001–1016. 10.1111/nph.12277 23594392

[B15] Dorofeev D. A. Artemenko E. N. Devyatkina G. A. (2001). Effect of metabolites from a *Fusarium* sp. on Fusarium root rot development in wheat plants. *Agro XXI* 10 12–13.

[B16] Felix G. Regenass M. Boller T. (1993). Specific perception of subnanomolar concentrations of chitin fragments by tomato cells. Induction of extracellular alkalinization, changes in protein phosphorylation, and establishment of a refractory state. *Plant J.* 4 307–316. 10.1046/j.1365-313X.1993.04020307.x

[B17] Fravel D. R. Bailey B. A. Bao J. (2003a). Differences in gene expression of pathogenic and biocontrol *Fusarium oxysporum*. *Phytopathology.* 93 S27.

[B18] Fravel D. R. Olivan C. Alabouvette C. (2003b). *Fusarium oxysporum* and its biocontrol. *New Phytol.* 157 493–502. 10.1046/j.1469-8137.2003.00700.x 33873407

[B19] Fravel D. R. Moravec B. C. Bailey B. A. (2007). Identification and regulation of genes from a biocontrol strain of *Fusarium oxysporum*. *J. Phytopathol.* 155 526–530. 10.1111/j.1439-0434.2007.01271.x

[B20] Fravel D. R. Moravec B. C. Jones R. W. Costanzo S. (2008). Characterization of two ABC transporters from biocontrol and phytopathogenic *Fusarium oxysporum*. *Physiol. Molecul. Plant Pathol.* 73 2–8. 10.1016/j.pmpp.2008.09.004

[B21] Freitas R. S. Steindorff A. S. Ramada M. H. S. de Siqueira S. J. L. Noronha E. F. Ulhoa C. J. (2014). Cloning and characterization of a protein elicitor Sm1 gene from *Trichoderma harzianum*. *Biotechnol. Lett.* 36 783–788. 10.1007/s10529-013-1410-4 24322765

[B22] Garcia-Brugger A. Lamotte O. Vandelle E. Bourque S. Lecourieux D. Poinssot B. (2006). Early signaling events induced by elicitors of plant defenses. *MPMI* 19 711–724. 10.1094/MPMI-19-0711 16838784

[B23] Garg A. Raghava G. P. S. (2008). ESLpred2: improved method for predicting subcellular localization of eukaryotic proteins. *BMC Bioinform.* 9:503 10.1186/1471-2105-9-503 PMC2612013 19038062

[B24] Hanson L. E. Howell C. R. (2004). Elicitors of plant defense responses from biocontrol strains of *Trichoderma virens*. *Phytopathology.* 94 171–176. 10.1094/PHYTO.2004.94.2.171 18943540

[B25] Hermosa R. Viterbo A. Chet I. Monte E. (2012). Plant-beneficial effects of *Trichoderma* and of its genes. *Microbiology* 158 17–25. 10.1099/mic.0.052274-0 21998166

[B26] Houterman P. M. Cornelissen B. J. C. Rep M. (2008). Suppression of plant resistance gene-based immunity by a fungal effector. *PLoS Pathog.* 4:e1000061 10.1371/journal.ppat.1000061 PMC2330162 18464895

[B27] Houterman P. M. Ma L. van Ooijen G. de Vroomen M. J. Cornelissen B. J. Takken F. L. (2009). The effector protein Avr2 of the xylem-colonizing fungus *Fusarium oxysporum* activates the tomato resistance protein I-2 intracellularly. *Plant J.* 58 970–978. 10.1111/j.1365-313X.2009.03838.x 19228334

[B28] Houterman P. M. Speijer D. Dekker D. L. de Koster C. G. Cornelissen B. J. Rep M. (2007). The mixed xylem sap proteome of *Fusarium oxysporum*-infected tomato plants. *Mol. Plant Pathol.* 8 215–221. 10.1111/j.1364-3703.2007.00384.x 20507493

[B29] Islam A. (2006). Fungus resistant transgenic plants: strategies, progress and lessons learnt. *Plant Tissue Cult. Biotechnol.* 16 117–138. 10.3329/ptcb.v16i2.1113

[B30] Jones D. T. Taylor W. R. Thornton J. M. (1992). The rapid generation of mutation data matrices from protein sequences. *Comput. Appl. Biosci.* 8 275–282. 1633570 10.1093/bioinformatics/8.3.275

[B31] Kaur R. Kaur J. Singh R. S. (2010). Nonpathogenic *Fusarium* as a biological control agent. *Plant Pathol. J.* 9 79–91. 10.3923/ppj.2010.79.91

[B32] Kromina K. A. Dzhavakhiya V. G. (2006). Expression of bacterial gene *CspD* in tobacco plants results in enhanced resistance to fungal and viral phytopathogens. *Mol. Gen. Virol. Microbiol.* 1 31–34. 16512609

[B33] Larkin R. P. Fravel D. R. (1999). Mechanisms of action and dose-response relationships governing biological control of *Fusarium* wilt of tomato by nonpathogenic *Fusarium* spp. *Phytopathology* 89 1152–1161. 10.1094/PHYTO.1999.89.12.1152 18944639

[B34] Larkin R. P. Fravel D. R. (2002). Reduction of *Fusarium wilt* of hydroponically-grown basil by *Fusarium oxysporum* strain CS-20. *Crop Prot.* 21 539–543. 10.1016/S0261-2194(01)00143-0

[B35] Larkin R. P. Fravel D. R. Everts K. L. (1999). Field efficacy of selected nonpathogenic *Fusarium* spp., and other biocontrol agents for the control of *Fusarium* wilt of muskmelon. *Biol. Cult. Tests* 161 1997–1998.

[B36] Lorang J. M. Sweat T. A. Wolpert T. J. (2007). Plant disease susceptibility conferred by a “resistance” gene. *Proc. Natl. Acad. Sci. U.S.A.* 104 14861–14866. 10.1073/pnas.0702572104 17804803 PMC1976202

[B37] Mandeel Q. Baker R. (1991). Mechanisms involved in biological control of *Fusarium* wilt of cucumber with strains of nonpathogenic *Fusarium oxysporum*. *Phytopathology* 81 462–469. 10.1094/Phyto-81-462

[B38] Mishura A. A. Sharma K. Misra R. S. (2009). Purification and characterization of elicitor protein from *Phytophthora colocasiae* and basic resistance in *Colocasia esculenta*. *Microbiol. Res.* 164 688–693. 10.1016/j.micres.2008.09.001 18990553

[B39] Mueller K. Chinchilla D. Albert M. Jehle A. K. Kalbacher H. Boller T. (2012). Contamination risks in work with synthetic peptides: flg22 as an example of a pirate in commercial peptide preparations. *Plant Cell* 24 3193–3197. 10.1105/tpc.111.093815 22923674 PMC3462625

[B40] Ökmen B. Etalo D. W. Joosten M. H. A. J. Bouwmeester H. J. de Vos R. C. H. Collemare J. (2013). Detoxification of α-tomatine by *Cladosporium fulvum* is required for full virulence on tomato. *New Phytol.* 198 1203–1214. 10.1111/nph.12208 23448507

[B41] Oliva R. Win J. Raffaele S. Boutemy L. Bozkurt T. O. Chaparro-Garcia A. (2010). Recent developments in effector biology of filamentous plant pathogens. *Cell. Microbiol.* 12 705–715. 10.1111/j.1462-5822.2010.01471.x 20374248

[B42] Olivain C. Humbert C. Nahalkova J. Fatehi J. L’Haridon F. Alabouvette C. (2006). Colonization of tomato root by pathogenic and non pathogenic *Fusarium oxysporum* strains inoculated together and separately into the soil. *Appl. Environ. Microbiol.* 72 1523–1531. 10.1128/AEM.72.2.1523-1531.2006 16461707 PMC1392888

[B43] Oomea S. Raaymakersa T. M. Cabrala A. Samwela S. Bohmc H. Albertc I. (2014). Nep1-like proteins from three kingdoms of life act as a microbe-associated molecular pattern in *Arabidopsis*. *Proc. Natl. Acad. Sci. U.S.A.* 111 16955–16969. 10.1073/pnas.1410031111 25368167 PMC4250136

[B44] Panina Y. Fravel D. R. Baker C. J. Shcherbakova L. A. (2007). Biocontrol and plant pathogenic *Fusarium oxysporum* induced changes in phenolic compounds in tomato leaves and roots. *J. Phytopathol.* 155 475–481. 10.1111/j.1439-0434.2007.01260.x

[B45] Paparu P. Dubois T. Coyne D. Viljoen A. (2007). Defense-related gene expression in (Musa spp.) following inoculation with non-pathogenic *Fusarium oxysporum* endophytes and challenge with *Radopholus similis*. *Physiol. Mol. Plant Pathol.* 71 149–157. 10.1016/j.pmpp.2007.12.001

[B46] Peng D. H. Qiu D. W. Ruan L. F. Zhou C. F. Sun M. (2011). Protein elicitor PemG1 from *Magnaporthe grisea* induces systemic acquired resistance (SAR) in Plants. *MPMI* 24 1239–1246. 10.1094/MPMI-01-11-0003 21770770

[B47] Pozo M. J. Azcon-Aguilar C. Dumas-Gaudot E. Barea J. M. (1998). Chitosanase and chitinase activities in tomato roots during interactions with arbuscular mycorrhizal fungi or *Phytophthora parasitica.* *J. Exp. Bot.* 49 1729–1739. 10.1093/jxb/49.327.1729

[B48] Recorbet G. Bestel-Corre G. Dumas-Gaudot E. Gianinazzi S. Alabouvette C. (1998). Differential accumulation of b-1,3-glucanase and chitinase isoforms in tomato roots in response to colonization by either pathogenic or non-pathogenic strains of *Fusarium oxysporum*. *Microbiol. Res.* 153 257–263. 10.1016/S0944-5013(98)80009-8

[B49] Rep M. van der Does H. C. Meijer M. van Wijk R. Houterman P. M. Dekker H. L. (2004). A small, cysteine-rich protein secreted by *Fusarium oxysporum* during colonization of xylem vessels is required for I-3-mediated resistance in tomato. *Mol. Microbiol.* 53 1373–1383. 10.1111/j.1365-2958.2004.04177.x 15387816

[B50] Rooney H. C. van’t Klooster J. W. van der Hoorn R. A. Joosten M. H. Jones J. D. de Wit P. J. (2005). Cladosporium Avr2 inhibits tomato Rcr3 protease required for CF2-dependent disease resistance. *Science* 308 1783–1786. 10.1126/science.1111404 15845874

[B51] Rotblat B. Enshell-Seijffers D. Gershoni J. M. Schuster S. Avni A. (2002). Identification of an essential component of the elicitation active site of the EIX protein elicitor. *Plant J.* 32 1049–1055. 10.1046/j.1365-313X.2002.01490.x 12492845

[B52] Ruocco M. Lanzuise S. Lombardi N. Woo S. L. Vinale F. Marra R. (2015). Multiple roles and effects of a novel *Trichoderma hydrophobin*. *MPMI* 28 167–179. 10.1094/MPMI-07-14-0194-R 25317667

[B53] Ryazantsev D. Y. Rogozhin E. A. Dimitrieva T. V. Drobyazina P. E. Khadeeva N. V. Egorov T. A. (2014). A novel hairpin-like antimicrobial peptide from barnyard grass (*Echinochloacrus galli* L.) seeds: structure-functional and molecular genetics characterization. *Biochimie* 99 63–70. 10.1016/j.biochi.2013.11.005 24275143

[B54] Sanger F. Nichlen S. Coulson A. R. (1977). DNA sequencing with chain-terminating inhibitors. *Proc. Natl. Acad. Sci. U.S.A.* 74 5463–5467. 10.1073/pnas.74.12.5463 271968 PMC431765

[B55] Seidl V. Marchetti M. Schandl R. Allmaier G. Kubicek C. P. (2006). Epl1, the major secreted protein of *Hypocrea atroviridis* on glucose, is a member of a strongly conserved protein family comprising plant defense response elicitors. *FEBS J.* 273 4346–4359. 10.1111/j.1742-4658.2006.05435.x 16939625

[B56] Shcherbakova L. A. Nazarova T. A. Mikityuk O. D. Fravel D. R. (2011). *Fusarium sambucinum* isolate FS-94 induces resistance against *Fusarium wilt* of tomato via activation and priming of a salicylic acid-dependent signaling system. *Russ. J. Plant Physiol.* 58 808–818. 10.1134/S1021443711050207

[B57] Silva J. C. Bettiol W. (2005). Potential of non-patogenic *Fusarium oxysporum* isolates for control of *Fusarium wilt* of tomato. *Fitopatol. Brasil.* 30 409–412. 10.1590/S0100-41582005000400012

[B58] Stassen J. H. M. Seidl M. F. Vergeer P. W. J. Nijman I. J. Snel B. Cuppen E. (2012). Effector identification in the lettuce downy mildew *Bremia lactucae* by massively parallel transcriptome sequencing. *Mol. Plant Pathol.* 13 719–731. 10.1111/j.1364-3703.2011.00780.x 22293108 PMC6638827

[B59] Stergiopoulos I. de Wit P. J. (2009). Fungal effector proteins. *Annu. Rev. Phytopathol.* 47 233–263. 10.1146/annurev.phyto.112408.132637 19400631

[B60] Stergiopoulos I. Kourmpetis Y. A. I. Slot J. C. Bakker F. T. de Wit P. J. G. M. Rokas A. (2012). In silico characterization and molecular evolutionary analysis of a novel superfamily of fungal effector proteins. *Mol. Biol. Evol.* 29 3371–3384. 10.1093/molbev/mss143 22628532

[B61] Takenaka S. Nishio Z. Nakamura Y. (2003). Induction of defense reactions in sugar beet and wheat by treatment with cell wall protein fractions from the mycoparasite *Pythium oligandrum*. *Phytopathology* 93 1228–1232. 10.1094/PHYTO.2003.93.10.1228 18944321

[B62] Takken F. Rep M. (2010). The arms race between tomato and *Fusarium oxysporum*. *Mol. Plant Pathol.* 11 309–314. 10.1111/j.1364-3703.2009.00605.x 20447279 PMC6640361

[B63] Tamura K. Peterson D. Peterson N. Stecher G. Nei M. Humar S. (2011). MEGA5: molecular evolutionary genetics analysis using maximum likelihood, evolutionary distance, and maximum parsimony methods. *Mol. Biol. Evol.* 28 2731–2739. 10.1093/molbev/msr121 21546353 PMC3203626

[B64] Thakur M. Sohal B. S. (2013). Role of elicitors in inducing resistance in plants against pathogen infection: a review. *ISRN Biochemistry* 2013:10 10.1155/2013/762412 PMC4393000 25969762

[B65] Thompson S. E. Smith M. Wilkinson M. C. Peek K. (2001). Identification and characterization of a chitinase antigen from *Pseudomonas aeruginosa* strain 385. *Appl. Environ. Microbiol.* 67 4001–4008. 10.1128/AEM.67.9.4001-4008.2001 11525997 PMC93121

[B66] Veloso J. Diaz J. (2012). *Fusarium oxysporum* Fo47 confers protection to pepper plants against *Verticillium dahliae* and *Phytophthora capsici*, and induces the expression of defense genes. *Plant Pathol.* 6 281–288. 10.1111/j.1365-3059.2011.02516.x

[B67] Wang B. Yang X. Zeng H. Liu H. Zhou T. Tan B. (2012). The purification and characterization of a novel hypersensitive-like response-inducing elicitor from *Verticillium dahliae* that induces resistance responses in tobacco. *Appl. Microbiol. Biotechnol.* 93 191–201. 10.1007/s00253-011-3405-1 21691787

[B68] Wang Y. Song J. Z. Wu Y. J. Odeph M. Liu Z. H. Howlett B. J. (2013). Eplt4 proteinaceous elicitor produced in *Pichia pastoris* has a protective effect against *Cercosporidium sofinum* infections of soybean leaves. *Appl. Biochem. Biotechnol.* 169 722–737. 10.1007/s12010-012-0015-z 23271623

[B69] Zhang Y. Yang X. Liu Q. Qiu D. Zhang Y. Zeng H. (2010). Purification of novel protein elicitor from Botrytis cinerea that induce disease resistance and drought tolerance in plants. *Microbiol. Res.* 165 142–151. 10.1016/j.micres.2009.03.004 19616421

