# Supplementary material for: Identification of a Novel Small Cysteine-Rich Protein in the Fraction from the Biocontrol Fusarium oxysporum Strain CS-20 that Mitigates Fusarium Wilt Symptoms and Triggers Defense Responses in Tomato
Source: Front Plant Sci. 2016 Jan 7;6:1207. doi: 10.3389/fpls.2015.01207 (PMC4703993; doi:10.3389/fpls.2015.01207)
Supplement: Supplementary file 1 [file Data_Sheet_1.PDF]

Supplementary Material

Identification of a novel small cysteine-rich protein in the fraction from the biocontrol *Fusarium oxysporum* strain CS-20 that mitigates Fusarium wilt symptoms and triggers defense responses in tomato

Larisa Shcherbakova*, Tatyana Odintsova, Alexander Stakheev, Deborah Fravel and Sergey Zavriev

*** Correspondence:** Larisa Shcherbakova: larisa@vniif.ru

		1 ... 23
F.avenaceum_(KIL89835)	(1)	MKFTTLATALFTLGLGADMAAAA
F.graminearum_PH-1_(XP_011325618)	(1)	MKFTTLATTILSITLGADLASAA
F.langsethiae_(KPA44764)	(1)	MKFTTLATTLLSITLGADIASAA
F.fujikuroi_IMI58289_CCT71502	(1)	MKFTALATAIFALGFGAEMATAA
F.verticillioides_7600_(EWG52846)	(1)	MKFTALATAIFALSFGAETATAA
F.oxysporum_Fo5176_(EGU83509)	(1)	MKLTTLATTIFALGFGADLATAA
F.oxysporum_f.sp.cubense_(EMT72127)	(1)	MKFTTIAATLLTLGFGADIAAAA
F.oxysporum_f.sp.radicis-lycopersici_(EXL42785)	(1)	MKFTTLAATLLTLGLGADIAAAA
F.oxysporum_Fo47_(EWZ33960)	(1)	MKFTTLSATLLTLGLGADIAAAA

Supplementary Figure 1. Alignment of signal peptide sequences of CS20EP homologs deposited in GenBank. Fully and partially identical amino-acid residues are highlighted by grey.
